# Occupational COVID-19 Prevention among Congolese Healthcare Workers: Knowledge, Practices, PPE Compliance, and Safety Imperatives

**DOI:** 10.3390/tropicalmed6010006

**Published:** 2020-12-30

**Authors:** Nzaji Michel-Kabamba, Nlandu Roger Ngatu, Ngombe Leon-Kabamba, Astrid Katumbo-Mukemo, Olivier Mukuku, Jean Ngoyi-Mukonkole, Guillaume Ngoie-Mwamba, Elie Kilolo-Ngoie, Ignace Bwana-Kangulu, Dora Kafusthi-Mukemo, Deca Blood Banza-Ndala, Denis Kabila-Mutombo, Marie-Claire Balela-Kabasu, Moise Kanyiki-Katala, Al Hassan Syed-Mahfuz, Akitsu Murakami, Kanae Kanda, Yukinori Mashima, Numbi Oscar-Luboya, Tomohiro Hirao

**Affiliations:** 1 Department of Public Health, Faculty of Medicine, University of Kamina, Kamina 279, Democratic Republic of the Congo; michelnzaji@yahoo.fr (N.M.-K.); leonkab@hotmail.com (N.L.-K.); guillaumengoiemwamba@gmail.com (G.N.-M.); eliekilolongoyumba@gmail.com (E.K.-N.); bwanaignace@yahoo.fr (I.B.-K.); oscarluboya@yahoo.fr (N.O.-L.); 2 Department of Public Health, Kagawa University Faculty of Medicine, Miki-cho 761-0793, Japan; mahfuz@med.kagawa-u.ac.jp (A.H.S.-M.); akitsu@med.kagawa-u.ac.jp (A.M.); oda@med.kagawa-u.ac.jp (K.K.); ymashima@med.kagawa-u.ac.jp (Y.M.); sharks@med.kagawa-u.ac.jp (T.H.); 3 Department of Public Health, Technical Medical College (ISTM), Lubumbashi 4748, Democratic Republic of the Congo; astridkatumbo081@gmail.com (A.K.-M.); oliviermukuku@yahoo.fr (O.M.); ngoyijean0@gmail.com (J.N.-M.); kafutshidora@yahoo.com (D.K.-M.); 4 Department of Epidemiology and Public Health, Technical Medical College (ITSM) of Mbuji-Mayi, Mbuji-Mayi 1244, Democratic Republic of the Congo; banzadeca2@gmail.com (D.B.B.-N.); dcmkabila@gmail.com (D.K.-M.); balelamarieclaire@gmail.com (M.-C.B.-K.); kanyikikatala@gmail.com (M.K.-K.); 5 Faculty of Medicine, University of Lubumbashi, Lubumbashi 1825, Democratic Republic of the Congo

**Keywords:** COVID-19, Democratic Republic of the Congo, health care workers, infection prevention, personal protective equipment

## Abstract

The SARS-CoV-2 (COVID-19) pandemic has had a tremendous impact on the functionality of health systems and world affairs. We assessed knowledge, attitudes, and practices (KAPs) of healthcare workers (HCWs) in the Democratic Republic of the Congo (DRC). This was a cross-sectional study conducted in 23 referral hospitals located in three towns of the DRC (Lubumbashi, Kamina, Mbuji-Mayi). In total, 613 HCWs were surveyed using the World Health Organization’s (WHO’s) “Exposure Risk Assessment in the Context of COVID-19” questionnaire. Participants included medical doctors (27.2%) and other categories of HCWs (72.8%). The mean age was 40.3 ± 11.7 years. Over 80% (range: 83–96%) of respondents had sufficient knowledge on each of the three domains: COVID-19 symptoms, disease transmission, and patient care approach. However, attitudes and practices scores were relatively low. Only 27.7% of HCWs were willing to receive a COVID-19 vaccine when it is available, whereas 55% of HCWs complied with good practices; 49.4% wore masks consistently and, surprisingly, only 54.9% used personal protective equipment (PPE) consistently at work and during contact with patients. Knowledge level was positively associated with the use of social media as a primary source of COVID-19-related information and the category of residence, with HCWs from towns already affected by the COVID-19 epidemic being more likely to have positive attitudes (adjusted OR, 1.64; 95%CI, 1.32–2.20) and comply with good practices (aOR, 2.79; 95%CI, 1.93-4.06). This study showed that most Congolese HCWs had sufficient knowledge on COVID-19, whereas the majority did not comply with consistent PPE use. The government of the DRC should urgently take major steps in capacity building for HCWs in outbreak preparedness and supplying hospitals with PPE.

## 1. Introduction

The ongoing SARS-CoV-2 (COVID-19) outbreak is tremendously impacting world affairs, with changes in the organization, functionality, and implementation of safety measures in healthcare settings. The first cases of COVID-19, a disease caused by the Severe Acute Respiratory Syndrome Coronavirus 2 (SARS-CoV-2), were reported on 8 December 2019 in Wuhan, China. Then, it spread outside Mainland China to become a global public health emergency [1,2]. The World Health Organization (WHO) designated COVID-19 a pandemic on 11 March 2020 [3]. Since then, the number of cases has increased in numerous countries across the globe, including among members of the healthcare workforce. As of 11 May 2020, there were 4,013,728 confirmed COVID-19 cases globally, including 278,993 deaths [4].

The African region remains the least-affected continent, with 43,909 cases and 1764 deaths, but the numbers are increasing. In the Democratic Republic of the Congo (DRC), the first COVID-19 case was reported on 10 March 2020 [4]. According to the latest report from the DRC’s COVID-19 Taskforce and Ministry of Health, the numbers of infected people in the DRC reached 1102 on 11 May 2020, including 44 deaths [5]. Amidst the pandemic, frontline healthcare workers (HCWs) play a crucial role in caring for COVID-19 patients and helping to counter the spread of the outbreak. However, several reports show evidence of the risk that they are facing, mainly due to the workload and the shortage of personal protective equipment (PPE). Obviously, the inadequate PPE supply is worrisome, as it puts HCWs at high risk for contracting a COVID-19 infection [3,6].

At the early phase of the outbreak, the WHO and Centers for Disease Control and Prevention (CDC) published recommendations for the prevention and control of COVID-19 for HCWs [7]. Additionally, the WHO provided online training sessions and materials on COVID-19 in various languages to strengthen preventive strategies, including raising awareness and training HCWs in preparedness activities [8]. On the other hand, poor understanding of the disease among HCWs may result in delayed diagnosis and treatment, leading to rapid spread of the infection. In March 2020, Italy reported that over 2600 HCWs were infected with COVID-19 [9,10]. Moreover, over 100 HCWs have lost their lives to COVID-19, which might be a barrier to fighting against the disease. It is noteworthy that inconsistent or lack of use of PPE and inadequate physical distancing are the main factors that expose HCWs to COVID-19 [11,12].

Healthcare-associated infections are reported to be a serious issue, as they are a common cause of morbidity and mortality not only among HCWs, but also among hospitalized patients [13]. Standard precautions are safety guidelines for the prevention of occupational infections occurring in health settings among members of the healthcare workforce; compliance with those guidelines, which includes the efficient use of appropriate PPE, has been shown to reduce the risk of exposure or contact with patients’ body fluids [14,15]. This study aimed to assess the knowledge, attitudes, and practices towards COVID-19 among Congolese HCWs from referral hospitals in Lubumbashi, Mbuji-Mayi, and Kamina in the DRC.

## 2. Materials and Methods

### 2.1. Study Design, Sites, and Participants

**Figure 1:**
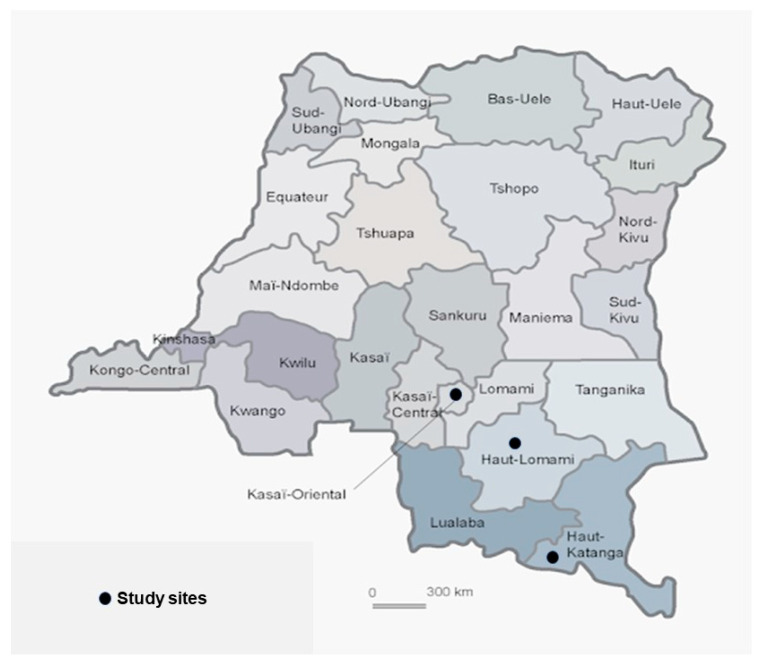
Map of Africa and the Democratic Republic of the Congo (source: Wikimedia).

A cross-sectional study was conducted in 23 Congolese referral hospitals, including three university hospitals, located in three towns in the DRC from 20 March through April 2020: seven in Lubumbashi (Haut-Katanga province), nine in Mbuji-Mayi (Kasai Oriental province), and seven in Kamina (Haut-Lomami province) (Figure 1). HCWs (doctors, nurses, midwives, laboratory technicians) aged 18 years or older who accepted to participate and worked in referral hospitals located in the above-mentioned towns were eligible for this study. Younger (aged less than 18 years) HCWs and those who were absent on the day of the survey were excluded.

The Figure shows the map of DRC with the three provinces where the study sites are located (Kasai-oriental, Haut-Katanga, and Haut-Lomami provinces).

### 2.2. Survey Questionnaire and Data Collection

Data were collected with the use of a self-administered questionnaire, which was derived from the “Exposure Risk Assessment in the Context of COVID-19” [16], in the French language, and consisted of two parts: demographics and knowledge, attitudes, and practices (KAPs). Demographic variables included age, gender, marital status, years of working experience, and sources of information on COVID-19. The second part included 12 questions on COVID-19-related knowledge (clinical manifestations, disease transmission, patient care approach), and three others on attitudes towards COVID-19 (confidence about overcoming the pandemic, willingness to get a COVID-19 vaccine); the last three questions were related to practices towards COVID-19 prevention (social distancing, general preventive measures, consistency of PPE use); each correct answer weighed one point (0 points for an incorrect answer).

Using Bloom’s cut-off point of 80% [17], a participant who provided correct answers to 80–100% of the 12 knowledge-related questions was considered to have sufficient COVID-19 knowledge. Regarding attitudes and practices, a participant with a score higher than the mean score was considered to have positive attitudes or to comply with good practices.

Enrollment of participants took place at each service site that provided patient care and each medical laboratory unit of the participating hospitals. Each participant received an anonymous questionnaire sheet after being informed of the objectives and activities of this study.

### 2.3. Ethical Approval

Verbal consent was obtained from each participant prior to being enrolled and answering the survey questionnaire. The study is part of the “Occupational Safety of Congolese Health care Workers Research” project, whose protocol was approved by the ethics committee of the School of Public Health of the University of Lubumbashi, DRC (approval letter No. UNILU/CEM/087/2019).

### 2.4. Statistical Analysis

Data were analyzed using IBM SPSS software version 23.0 (IBM Corporation, Armonk, NY, USA). Categorical data related to answers to questions about knowledge, attitudes, and practices towards COVID-19 are presented as frequencies and proportions. On the other hand, data related to continuous variables are summarized as means with standard deviations or ranges. Chi-squared and unpaired *t* tests were used to compared subgroups of HCWs. A multivariate logistic regression model was employed to determine associations between dependent (KAP) and independent (demographic) variables. Statistical difference was set at a *p*-value of less than 0.05.

## 3. Results

### 3.1. Sociodemographic Characteristics of the Participants

Of the 752 HCWs (202 from Mbuji-Mayi, 370 from Lubumbashi, and 180 from Kamina) who received the survey questionnaire, there were 613 respondents (85.6% response rate). Most of the respondents (54.5%) were working in hospitals located in Lubumbashi in the Haut-Katanga province, followed by HCWs from Mbuji-Mayi in the Kasai-oriental province (28.9%) and the town of Kamina in the Haut-Lomami province (16.6%). Women accounted for 49.1% of the respondents. A total of 27.2% were doctors, whereas the remaining HCWs (72.8%) included nurses, midwives, and laboratory technicians (Table 1).

The mean age was 40.3 ± 11.7 years; most of the respondents (95.3%) were 25 years or older. The majority of respondents were married (66.6%), had less than 10 years of working experience (53.5%), and had already heard about COVID-19 (99.3%); however, only 41.9% of them had attended a lecture, meeting, or discussion about the disease (Table 1).

### 3.2. Knowledge of Congolese Health Care Workers about COVID-19

Overall, 83% of respondents had sufficient knowledge about common clinical symptoms of COVID-19 (89.2% of doctors vs. 80.7% of other HCWs; *p* < 0.05), and 85% had sufficient knowledge of COVID-19 patient care (89.8% of doctors vs. 83.8% of other HCWs; *p* < 0.05). Regarding knowledge on the disease’s transmission, over 90% of the respondents had sufficient knowledge: 92.5% for the spread through respiratory droplets (95.8% of doctors vs. 91.3% of other HCWs), 96.5% and 93.5% for isolation of patients and their contacts, respectively, and 92.3% for avoidance of crowded places.

Similarly, a higher proportion of the respondents (73.1%) had sufficient knowledge on risk factors for severe COVID-19 infection, including 71.9% of doctors and 73.5% of other HCWs, whereas only 46.7% had insufficient knowledge about clinical differential diagnosis between COVID-19 and other respiratory diseases (Table 2).

**Figure 2:**
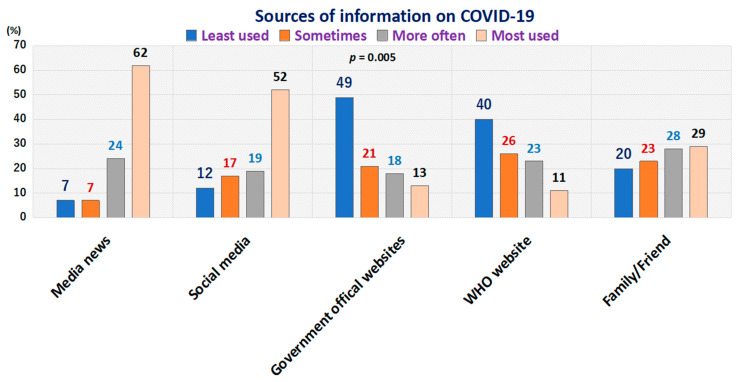
Distribution of respondents (%) according to the frequency of use of sources of information on COVID-19.

Participants were also asked about their primary source of information on COVID-19; it was observed that most of the respondents mostly used the news media and social media as primary sources of information on COVID-19, whereas the government’s and WHO’s websites were used less, as shown in Figure 2.

The figure shows that most of the respondents used the news media and social media to get information on COVID-19, whereas they used the government’s and WHO’s official COVID-19-related websites less.

### 3.3. Attitudes and Practices of Health Care Workers towards COVID-19

**Figure 3:**
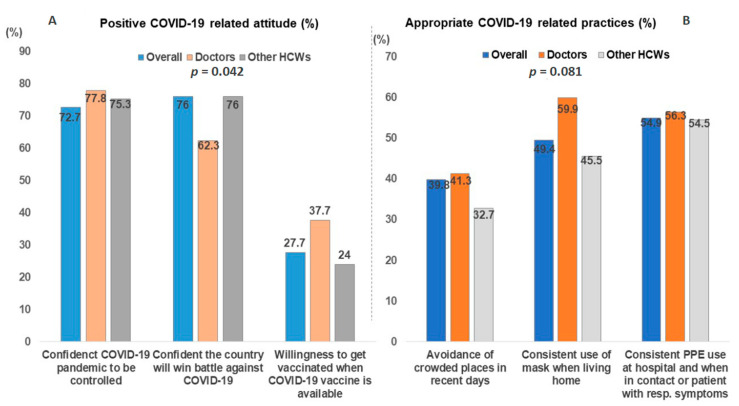
Attitudes and practices of the respondents towards COVID-19. (**A**) Proportions of participants with positive COVID-19-related attitudes; (**B**) Proportions of participants with appropriate COVID-19-related practices

The mean attitude score was 1.7 ± 0.9 out of 3; the questions were mainly related to confidence on the success of the COVID-19 outbreak response in the DRC and the willingness for HCWs to get vaccinated with an anti-COVID-19 vaccine. The majority (76%) of the respondents agreed that COVID-19 will be successfully controlled (77.8% of doctors vs. 75.3% of other HCWs); on the other hand, 72.3% (62.3% of doctors vs. 76% of other HCWs; *p* < 0.05) were confident that the DRC can win the battle against COVID-19. However, only 27.7% of the respondents answered that they were willing to get vaccinated with a COVID-19 vaccine if it was available, including 37.7% of doctors vs. 24% of other HCWs (*p* < 0.05) (Figure 3A).

The mean practices score was relatively low, with 1.6 ± 0.8 out of 3; only 55% of HCWs complied with good practices, and 64.9% of respondents reported avoiding crowded places (58.7% of doctors vs. 67.3% of other HCWs). Less than half (49.4%) were wearing masks consistently (59.9% of doctors vs. 45.5% of other HCWs). Regarding PPE use at work, almost half (54.9%) of the respondents used them consistently (56.3% of doctors vs. 54.5% of other HCWs) during contact with or when caring for patients with respiratory symptoms (Figure 3B).

The figure shows that a relatively large proportion of the respondents had positive attitudes towards COVID-19 in regards to confidence about the control of the COVID-19 pandemic, whereas a large majority was not ready to accept being vaccinated with a COVID-19 vaccine (Figure 3A). On the other hand, a large proportion of the respondents did not comply with preventive measures regarding avoidance of crowded places (39.8%) and the use of masks (49%), and only about half of them used PPE consistently (Figure 3B).

### 3.4. KAPs’ Determinants among Congolese Healthcare Workers

Table 3 shows the relationship between KAPs and the characteristics of the respondents. Multivariate logistic regression analysis showed that COVID-19 knowledge was positively associated with the use of social media (aOR: 1.69 ± 0.56; 95%CI: 1.87-3.25; *p* < 0.01) and family members/friends (aOR: 1.87 ± 0.79; 95%CI: 1.82–4.30) as sources of primary information on COVID-19, as well as COVID-19-related practices (aOR: 3.45 ± 2.40; 95%CI: 1.88–13.49; *p* < 0.05). COVID-19-related attitudes were associated with the category of residence (aOR: 1.64 ± 0.17; 95%CI: 1.32–2.2; *p* < 0.05) and COVID-19 knowledge (aOR: 2.36 ± 0.74; 95%CI: 1.27–4.39; *p* < 0.01). COVID-19-related practices were associated with the use of social media (aOR: 1.43 ± 0.24); 95%CI: 1.01–2.02; *p* < 0.05) and category of residence (aOR: 2.79 ± 0.28; 95%CI: 1.93–4.06); *p* < 0.01) (Table 3).

## 4. Discussion

The present study is the first to assess the risk of exposure for the Congolese HCWs in the context of the COVID-19 pandemic. It was conducted in 23 referral hospitals located in three provinces of the DRC, a country that is facing increasing morbidity and mortality due to this disease. The ongoing COVID-19 outbreak is considered an emergency, and healthcare providers are seen to have an increased risk of infection. The pandemic is an emerging, rapidly changing global health challenge that is affecting all sectors [1], and the likelihood of acquiring the disease is higher among HCWs compared to the general population [18]. Thus, it is of paramount importance that HCWs should have accurate knowledge on all aspects of the disease, including the symptoms, diagnosis, proposed treatment, and prevention strategies, and should display necessary safety practices in order to prevent contamination and the spread of the infection.

In this study, it was observed that the respondents relied on social media, family, and friends for information on COVID-19 and had sufficient knowledge on the disease. Similar trends have recently been reported in a Ugandan study [17], where a higher proportion (69%) of participants knew about the disease, and a Chinese survey that found sufficient knowledge in 89% of HCWs [19]. The high knowledge score in Congolese HCWs has its roots partly in their high exposure to the information provided by the media about the virus since the start of the outbreak. This suggests that, in addition to improving medical help, media coverage can be considered as an effective way to mitigate the spread of the disease [20]. DRC officials should consider a variety of channels to update knowledge and learning materials about this epidemic, and especially to communicate information to those that are not aware of any issue relating to COVID-19.

Our study also showed that most of the respondents were confident that the COVID-19 pandemic would be successfully controlled both at the world level and in the DRC. However, despite this confidence, a large portion of HCWs have not been complying with recommended safety measures for limiting the spread of COVID-19. In fact, the practice score was very low, and almost half of the HCWs did not use PPE consistently or avoid crowded places for COVID-19 infection prevention. This situation is very alarming, as it suggests negligence by HCWs and poor work safety in Congolese health settings.

Previous studies have shown high incidence of occupational injury and exposure to blood and other body fluids (BBFs) occurring in several Congolese hospitals in the Katanga and Kongo central provinces, as well as the capital Kinshasa, mainly due to the limited provision of PPE and the lack of safety-engineered medical devices (SEDs) [21,22]. In fact, safety syringes, needles, catheters, etc. are still not in use in the majority of Congolese health settings, even in urban hospitals, exposing HCWs to the risk of contracting communicable infectious diseases. It is unacceptable that frontline care providers are either unprepared or lack necessary equipment to protect themselves and their patients from infection in a country that has been facing outbreaks of viral infections, such as the Ebola and Marburg epidemics [23,24] and, currently, COVID-19. A study conducted in Pakistan also showed that PPE was not available in many healthcare settings, and compliance with PPE use was very low among HCWs [25].

Obviously, with the COVID-19 outbreak, the shortage of PPE supplies has become a global problem. To prevent spread of the disease to and from HCWs and patients requires the availability and effective use of PPE; thus, in addition to training HCWs to acquire necessary skills and capacities for preventing the disease, the scarcity of PPE in healthcare settings at the time when it is needed the most to care for highly infectious patients should be addressed quickly and efficiently [3,25,26,27].

The loss of members of the health care workforce due to COVID-19 is worrisome. China, for example, has lost over 20 of the 3000 HCWs with COVID-19 [28], and a report from the Philippines’ government showed that over 3000 HCWs directly affected the outbreak, of whom 1080 were infected and 26 died by April 2020 [29].

Another informative finding from our study is that most HCWs (over 70%) were reluctant to receive a COVID-19 vaccine. This fact is in line with the general opinion in the sub-Saharan African countries due to rumors about an underlying motivation to use Africa as an experimental land for the vaccine, though the most severely affected countries are Western nations [30]. It is quite common to face social and cultural obstacles while responding to outbreaks in countries of the region. For example, our recent nationwide Ebola survey showed that there were residents who questioned the existence of Ebola in the eastern Congo during the 2020 outbreak, and others were reluctant towards receiving Ebola vaccine, saying it was a plot or a biological weapon used to make men sterile and depopulate the regions [31].

Furthermore, following a declaration in the media by a French professor on a COVID-19 vaccine trial on this continent, the African Centers for Disease Control and Prevention (Africa CDC) condemned the expert’s discriminatory comments [32]. In other parts of the African continent, the situation may differ; for example, an Egyptian study showed a large majority of the participants (73%) willing to be vaccinated once the anti-COVID-19 vaccine is available [33].

As a limitation, the cross-sectional design used in this study does not allow the generalization of the findings to the entire Congolese healthcare workforce. Nonetheless, this study provides a general view on the work safety issues in Congolese hospitals. Additionally, it is the first study to explore the risk factors and behaviors that might cause occupational exposure to COVID-19 among Congolese HCWs. Moreover, the relatively big sample size of participants and the large number of health settings that were involved in this study are other strengths of this study.

## 5. Conclusions

The present study provides a snapshot of the need for enhanced COVID-19 mitigation efforts in Congolese health settings. More importantly, it highlights information on the importance of compliance with recommended preventive measures, particularly with respect to the consistent use of appropriate PPE to counter the spread of the COVID-19 outbreak. The DRC’s government and health policymakers should urgently take major steps to train HCWs in emergency preparedness and response and to supply enough PPE, as the country is currently facing the spread of COVID-19 infection. Additionally, the implementation of periodic risk assessments and training for capacity building on “Standard Precautions” for occupational infection prevention in all Congolese health settings are also recommended.

## Figures and Tables

**Table 1:** Characteristics of the respondents (N = 613).

Variable	Participants (%)
**Gender**	
Male	312 (50.9)
Female	301 (49.1)
**Age**, mean ± SD	40.31 ± 11.67
<25 years	29 (4.7)
25–40 years	386 (63.0)
˃40 years	198 (32.3)
**Marital status**	
Married	408 (66.6)
Unmarried and widowed	205 (33.4)
**Occupation**	
Doctor	167 (27.2)
Nurses	332 (54.2)
Laboratory technician and medical biologist	58 (9.5)
Pharmacist	23 (3.8)
Administrator–manager	21 (3.4)
Dentist	8 (1.3)
Midwife	4 (0.7)
**Years of working experience**	
≤10	328 (53.5)
˃10	285 (46.5)
**Town of residence**	
Lubumbashi (Haut-Katanga province)	334 (54.5)
Kamina (Haut-Lomami province)	102 (16.6)
Mbuji-Mayi (Kasai-oriental province)	177 (28.9)
**Heard about novel coronavirus (SARS-CoV2)**	
Yes	609 (99.3)
No	4 (0.7)
**Attended lecture/discussion on Covid-19**	
Yes	257 (41.9)
No	356 (58.1)

Notes: SD: standard deviation; %: percentage.

**Table 2:** Distribution of proportions of correct answers regarding SARS-CoV-2 (COVID-19) knowledge.

Items	All HCWs	Doctors	Other HCWs	*p*
1. Common symptoms associated with COVID-19 infection	509 (83.0)	149 (89.2)	360 (80.7)	**0.013**
2. Clinical difference between COVID-19 and other lung disease symptoms	286 (46.7)	69 (41.3)	217 (48.7)	0.105
3. Currently, in absence of effective cure, symptomatic and supportive treatments help most patients to recover	521(85.0)	150 (89.8)	371 (83.8)	**0.041**
4. Not all infected persons develop severe disease; elderly and people with chronic conditions are likely to develop severe disease	448 (73.1)	120 (71.9)	328 (73.5)	0.675
5. Eating or getting in contact with wild animals as one risk factor for COVID-19 infection	277 (45.2)	91 (54.5)	186 (41.7)	**0.005**
6. In absence of fever, a person with COVID-19 may not transmit the virus to others	403 (65.7)	140 (83.8)	263 (59.0)	**0.000**
7. COVID-19 spreading via respiratory droplets of infected individuals	567 (92.5)	160 (95.8)	407 (91.3)	0.057
8. For ordinary residents, wearing general medical masks can prevent COVID-19 infection	481(78.5)	126 (75.4)	355 (79.6)	0.266
9. Strict adherence to COVID-19 prevention guidelines may not totally be applicable to children and young adults	180 (29.4)	40 (24.0)	140 (31.4)	0.072
10. Avoiding crowded places and public transportation as a way to prevent COVID-19 infection	566 (92.3)	155 (92.8)	411 (92.2)	0.784
11. Isolation and treatment of COVID-19-infected persons are effective ways to reduce the spread of the virus	590 (96.2)	164 (98.2)	426 (95.5)	0.119
12. People having contact with COVID-19 patient are to be isolated for at least 14 days.	573 (93.5)	159 (95.2)	414 (92.8)	0.287

Notes: HCWs: health care workers; *p*: *p*-value by chi-2 test.

**Table 3:** Determinants of knowledge, attitudes, and practices (KAPs) in HCWs according to multivariate logistic regression analysis.

Variables	Knowledge		Attitudes		Practices	
	aOR (SE)	95%CI	aOR (SE)	95%CI	aOR (SE)	95%CI
Gender (F vs. M)	0.45 (1.62)	0.22–0.91	0.65 (0.12)	0.46-0.93	1.03 (0.17)	0.74–1.44
Age (>40 y vs. 40 y or older)	1.08 (0.31)	1.01–1.14	1.02 (0.16)	0.99-1.06	1.01 (0.01)	0.99–1.03
Occupation (doctor vs. others)	0.97 (0.12)	0.76–1.24	0.82 (0.59)	0.72-0.94	0.85 (0.58)	0.74–0.97
Working experience	0.96 (0.31)	0.91–1.03	0.97 (0.16)	0.93–1.04	1.02 (0.15)	0.98–1.03
Category of residence (town without/with COVID-19 cases)	0.46 (0.93)	0.31–0.69	1.64 (0.18) #	1.32–2.05	2.79 (0.28) #	1.93–4.06
Information: social media	1.69 (0.56) *	1.87–3.25	1.15 (0.43)	0.86–2.63	1.43 (0.24) *	1.02–2.17
Information: family or friend	1.93 (0.74) #	2.90–4.11	0.89 (0.19)	0.58–1.36	1.71 (0.29) #	1.22–2.38
Information: official sites	2.17 (0.99)	0.88–5.34	0.81 (0.17)	0.54–1.21	1.31 (0.52)	0.59–2.86

Notes: *, *p* < 0.05; #, *p* < 0.01 (multivariate logistic regression analysis with adjustment for age and gender).

## Data Availability

Study data are available from the first author at the University of Kamina, DRC.
